# Post IVF heterotopic pregnancy with one in cervix and one in uterus. Successful delivery after termination of the cervical pregnancy with intraamniotic feticide

**DOI:** 10.1016/j.ijscr.2025.110832

**Published:** 2025-01-04

**Authors:** G. Michos, R. Najdecki, G. Valasoulis, A. Daponte, A. Mamopoulos, E.G. Papanikolaou

**Affiliations:** a Third Department of Obstetrics and Gynaecology, Aristotle University of Thessaloniki, Greece; b Assisting Nature, Centre of Reproduction and Genetics, Thessaloniki, Greece; c Obstetrics and Gynecology Department, University Hospital of Larisa, Greece

**Keywords:** Heterotopic pregnancy, Cervical pregnancy, Intraamniotic feticide, Case report

## Abstract

**Introduction:**

Cervical pregnancy is a rare kind of ectopic pregnancy. Heterotopic pregnancy is a condition, where we have one sac in the uterus and one in another location, usually because of IVF treatment. This scenario can become a life-threatening condition, if remain untreated.

**Presentation of case:**

A 44-year-old woman underwent IVF (egg donation) with double embryotransfer and resulted in twin pregnancy, however heterotopic. One in cervix and one intrauterine. Until 8 weeks both pregnancies evoluting equally and then a decision made to terminate the cervical one. Her physician chose a transabdominal approach (amniocentesis wise); however, this attempt failed. Then, intracervical puncture by a reproductive specialist was attempted with potassium chloride injection and aspiration of the amniotic fluid. The pregnancy was terminated successfully, and no complications presented afterwards. The intrauterine pregnancy evoluted normally and a livebirth was achieved at 39 weeks.

**Discussion:**

The current case represents an interesting way of terminating a cervical pregnancy even in the presence of a twin intrauterine sibling.

**Conclusion:**

Patients with heterotopic pregnancies, should be encouraged not to terminate both pregnancies and to be referred in specialized reproductive and fetal maternity centers.

## Highlights

• Heterotopic pregnancy is a rare condition
• This condition can become life-threatening
• Intracervical puncture and aspiration of the amniotic fluid of intracervical pregnancy is an effective treatment
• Patients with heterotopic pregnancies, should be encouraged not to terminate both pregnancies and to be referred in specialized reproductive and fetal maternity centers

## 1 Introduction

The simultaneous occurrence of an ectopic and intrauterine pregnancy is known as heterotopic pregnancy. Studies have shown that controlled ovarian stimulation and multiple embryo transfer during IVF are associated with an increased risk of heterotopic pregnancy [1]. One rare kind of ectopic pregnancy is called heterotopic cervical pregnancy (HCP). It is a situation in which there is at least one sac in the cervical canal and one gestational sac in the uterus [2]. One in every 30,000 pregnancies is said to have an incidence of HCP (once spontaneous pregnancy occurs). Nevertheless, this ratio is expected to be one in 100 in-vitro fertilization (IVF) patients due to the use of artificial reproductive techniques and it is associated with high morbidity and mortality [3]. Fertility preservation is the primary objective of the various strategies that have been described for the management of this kind of pregnancy. Methotrexate or transvaginal potassium chloride injections are examples of conservative treatment while cervical curettage with or without cerclage, and Foley catheter insertion are examples of surgical techniques [4]. Herein, we represent a minimally invasive method, ending an HCP even when there is an intrauterine twin sibling present yet.

This case report has been written in accordance with the SCARE criteria [5].

## 2 Presentation of the case

A 44-year-old woman, referred to our clinic after multiple failed IVF procedures. She agreed on egg donation and after proper matching she underwent endometrium preparation with hormone replacement therapy and double embryotransfer.

At 5 weeks a twin pregnancy was documented but unfortunately it was heterotopic with one sac in cervical os and one intrauterine. Since the possibility of vanishing twins is almost 20 %, they decided to follow the pregnancy hoping the cervical pregnancy (CP) to regress.

**Figs. 1-2:**
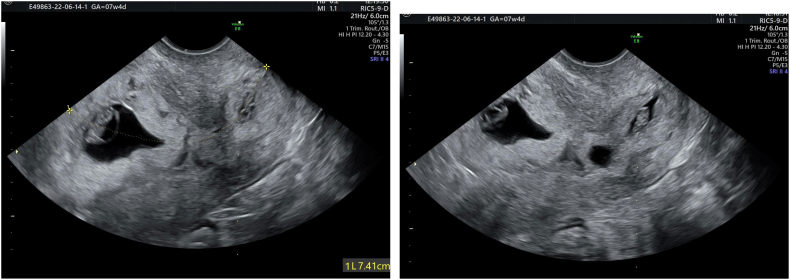
Heterotopic pregnancy, one sac intrauterine and one sac in the cervix.

Unfortunately, both sacs develop normally with heart activity becoming present in both fetuses. At 7 and 9 weeks, both embryos were viable with a CRL of 23 mm the cervical one and CRL of 25 the intrauterine one Figs. 1-2. At his point her obstetrician decided to proceed with elective feticide of the cervical fetus since methotrexate could not be administered in the presence of the intact intrauterine gestational sac. The transabdominal route was opted and at 9 + 2 weeks they tried unsuccessfully to proceed with the cervical pregnancy termination.

Then the patient was referred to our assisted reproduction clinic and a transvaginal approach was chosen to terminate the cervical ectopic, by a fertility specialist doctor. One ml of potassium chloride was mixed with 9 ml normal saline, and in the operating theater under mild sedation with the transvaginal probe of a S8 ultrasound (GE) we punctured intracervically the sac and then fetal body injecting 1 ml of KCL dilution near the heart. Once bradycardia was established, we retrograde gradually and aspirated also the amniotic fluid in order to establish complete anhydramnion of the gestational sac. Then we removed our needle, we controlled for 5 min for vaginal bleeding and the patient moved to her room.

The next day, the patient discharged after sonographic reassuring that the intrauterine embryo is intact and viable. Next days she underwent serial ultrasounds with no adverse outcome except experiencing mild vaginal bleeding. At 12th week patient underwent nuchal translucency scanning with normal measurements of the viable embryo and showing a small remnant of the ectopic sac on the cervix. Following weeks of pregnancy were uncomplicated, with scans and scheduled blood tests within normal limits.

At 39 weeks she delivered with cesarean section a healthy baby girl proving that the whole management was the appropriate.

## 3 Discussion

Prior to the 1980s, cervical pregnancy was difficulty diagnosed, usually by histology reports, after excessive bleeding caused by trophoblastic arteries eroded the cervix, necessitating a hysterectomy [6]. Advancements in ultrasound imaging have enabled early detection of cervical pregnancy, leading nowadays to more conservative treatment options. Ectopic cervical pregnancy is an uncommon type of ectopic pregnancy, accounting for fewer than 1 % of all pregnancies of unknown location [3].

The incidence of heterotopic pregnancy with a cervical component is even more uncommon. The high incidence of artificial reproductive techniques (ART) has resulted in an increase in the frequency of HCP, which was previously unusual [7]. The link is unclear; however, it could be explained by a combination of risk factors. There is a possible link between common risk factors for patients undergoing this procedure (e.g., cervical irregularities and history of previous curettage) and method-related factors (e.g., cervical trauma during the process, volume and viscosity of the transfer medium, and embryo reflux). Ultrasound-guided embryo transfer, though enhanced the number of successful pregnancies, seems to have no impact on the rate of heterotopic/ectopic pregnancy [8].

A cervical ectopic pregnancy can't proceed normally. To prevent life-threatening complications, the cervical pregnancy needs to be removed or ceased immediately after detection. Depending on serum β-HCG, weeks of pregnancy and whether the cervical one is live or not, recommendation is either medical treatment with drugs or surgical aspiration procedure.

Moragianni and colleagues [9] published a case series of 39 patients with HCP. Interestingly, 30 of them were IVF related. Most of them were managed surgically and only one case (*n* = 1/39, 3 %) resolved spontaneously.

Surgical excision of trophoblast was the preferred method for instant excision of trophoblast tissue in the past. Curettage is the age-old fertility preserving method, but with high hemorrhage risk. Primary hysterectomy is not a preferred method nowadays, unless the patient is presented in intractable hemorrhage, second trimester or third trimester diagnosis of CP and willing to avoid emergency surgery and blood transfusion in a woman with no future fertility plans. Only a few cases discuss second-trimester CEP management. All these cases of advanced CEP were linked with significant bleeding following placental detachment [10].

Recently, a combination of laparoscopy-assisted uterine artery ligation followed by hysteroscopic transcervical resection of CP has been described as a fertility-preserving alternative therapy [7]. We believe that such approach is unnecessary completely.

Systemic chemotherapy, with methotrexate, though popular for single ectopic pregnancies could not be an option in our case as there was an intrauterine viable sac and the pregnancy was desired.

The current case demonstrates how a conservative minimally invasive surgical procedure managed successfully HCP and even lead to a live birth. Ultrasound-guided intra-amniotic instillation of potassium chloride and/or methotrexate has been used as a conservative approach for the management of CP. Both these procedures require skill and expertise. We strongly believe that the gold-standard treatment of cervical pregnancy should be local transvaginal therapy. According to recent literature in 81 % of cases transvaginal therapy does not produce complications, in 5 % bleeding occurs requiring additional interventions and only in 1 % of cases is it necessary to remove the uterus [9].

## 4 Conclusion

Early pregnancy treatment is crucial, particularly for individuals with established infertility, as demonstrated in this case study. There are currently no guidelines or suggested first-line techniques for managing HCP due to a lack of published cases in international literature. Individualized treatment must consider the patient's goal to maintain the intrauterine pregnancy, as well as the medical team's experience and equipment.

In case of heterotopic pregnancies with cervical pregnancy, patients should be encouraged not to terminate both pregnancies and to be referred in specialized reproductive and fetal maternity centers.

## Author contribution

**Michos Georgios**: Paper design, data collection, paper writing, paper review.

**Najdecki Robert**: Paper design, data collection.

**Valasoulis Georgios**: Paper design, data collection.

**Daponte Alexandros**: Paper design, paper review.

**Mamopoulos Apostolos**: Paper design, paper review.

**Papanikolaou Evaggelos**: Paper design, paper review, picture preparation.

## Patient consent

Written informed consent was obtained from the patient for publication of this case report and accompanying images. A copy of the written consent is available for review by the Editor-in-Chief of this journal on request.

## Ethical approval

This article does not contain any personal information that can lead to the identification of the patient. As per local policy, Bioethics committee of Medical School, Faculty of Health Sciences, Aristotle University of Thessaloniki, case reports, apart from the informed consent of the patient, does not require ethical approval, as case reports or case series do not constitute research at our institution. Ethical approval is exempt/waived at our institution for this type of articles.

## Guarantor

Georgios Michos.

## Research registration number

N/A.

## Funding

None.

## Conflict of interest statement

None.
